# Review of Random Fiber Lasers for Optical Fiber Sensors

**DOI:** 10.3390/s23208500

**Published:** 2023-10-16

**Authors:** Meng Tian, Wentao Zhang, Wenzhu Huang

**Affiliations:** 1 State Key Laboratory of Transducer Technology, Institute of Semiconductors, University of Chinese Academy of Sciences, Beijing 100083, China; tianmeng@semi.ac.cn (M.T.); hwzhu@semi.ac.cn (W.H.); 2 Center of Materials Science and Optoelectronic Engineering, University of Chinese Academy of Sciences, Beijing 100049, China; 3 Shenzhen Academy of Disaster Prevention and Reduction, Shenzhen 518003, China

**Keywords:** random fiber laser, optical fiber sensor, light source, sensitive element

## Abstract

A random fiber laser does not need a traditional resonant cavity and only uses the multiple scattering of disordered media to provide feedback to achieve laser output. Therefore, it has the advantages of a simple structure, narrow linewidth, and low noise and is particularly suitable for fiber optic sensors. This paper provides an introduction to the categories and corresponding principles of random fiber lasers. The research progress of random fiber lasers in the sensing field in recent years, including various aspects of random fiber lasers as low-noise light sources or sensitive elements for fiber sensing systems, is the main focus. Finally, the future development trend of random fiber lasers for optical fiber sensors is explored.

## 1. Introduction

**Figure 1:**
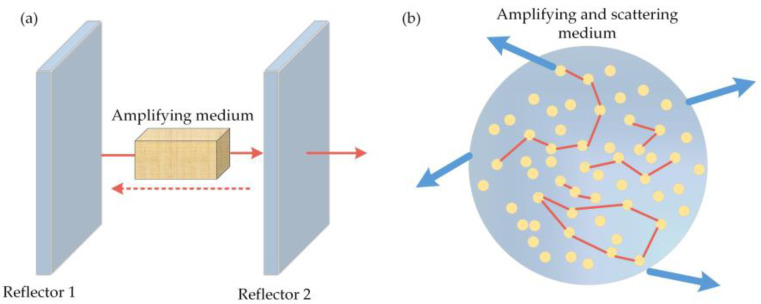
Comparison between the conventional laser (**a**) and random laser (**b**).

The proposed random laser (RL), as a new type of laser, has received much attention. The conventional laser consists of three parts: (i) a pump for power; (ii) an active medium for light amplification; and (iii) a resonant cavity for feedback to ensure a single mode or a small amount of axial mode oscillation. Therefore, the resonant cavity determines the key characteristics of the laser, such as the output spectrum, spatial mode structure, and directionality. In contrast, a random laser does not require a fixed optical resonant cavity and only utilizes the multiple scattering of photons in a disordered medium (Figure 1). The properties of the output laser are predominantly determined by the characteristics of the gain and the scattering medium.

The random laser was first discovered by R.V. Ambartsumyan and his colleagues [1]. By replacing the reflector of a ruby laser with a scattering surface and increasing the pump power, the emission spectrum gradually narrowed. Due to their special structure, RLs have attracted widespread attention and have been used in materials, such as semiconductor powders [2,3,4], liquid crystals [5,6,7], polymers [8,9,10,11], and biological tissues [12,13], to achieve laser emission. However, the above RLs are mostly two-dimensional or three-dimensional structures, and the directionality is poor. To control the direction of laser output, de Matos et al. [14] proposed a random fiber laser (RFL) that injected a gain liquid and nano-scattering particles into a one-dimensional medium, photonic crystal fiber (PCF), which effectively confined the laser within the fiber core. Unfortunately, this kind of RFL was prone to problems, such as poor long-term stability, complex structure, and difficulty in compatibility with fiber optic systems, hindering its development. Based on this, Turitsyn [15] directly utilized the Rayleigh scattering caused by the nonuniformity of the core material of a standard single-mode fiber (SMF) to provide feedback and nonlinear effects, such as stimulated Raman scattering (SRS) or stimulated Brillouin scattering (SBS), to provide gain; this achieved an all-fiber random laser, which is called a Rayleigh scattering random fiber laser (RS-RFL). RS-RFL fundamentally overcame the shortcomings of traditional RL, and it was a major breakthrough based on the concept of RL and greatly promoted the development process of RFL. In addition, N Lizárraga et al. [16] proposed another type of random fiber laser, called the grating feedback random fiber laser; this laser reduced the length of the feedback medium to tens of centimeters by writing several randomly spaced weak fiber gratings on erbium/germanium (Er/Ge) co-doped fibers.

Since RFL was proposed, many researchers have explored the physical mechanism, output characteristics, and implementation methods of RFL. RFL has been well applied in fields such as supercontinuum spectroscopy [17,18,19,20], speckle free imaging [21,22,23,24,25,26], fiber communication [27,28,29,30,31,32,33,34,35,36], and fiber sensing [37,38,39,40,41,42,43,44,45,46,47,48,49,50]. Especially in the field of fiber optic sensing, RFL exhibits significant advantages in fiber optic sensing systems due to its simple structure, narrow linewidth, and low noise. When used as the light source of a fiber optic sensing system, RFL can reduce the system spurious signal and noise and improve the signal-to-noise ratio (SNR) and detection distance; when used as the sensing element, it can improve the sensitivity and resolution of the system, etc. This paper mainly provides a review of the applications of random fiber lasers in the sensing field in recent years, including RFLs as light sources and sensing components.

Based on the different media providing feedback, the research progress of Rayleigh scattering RFL and grating feedback RFL in the sensing field is the main focus of this review. The layout is as follows: Section 1 mainly introduces the development history of RFLs and their advantages in the field of fiber sensing. Section 2 describes the research progress of RFLs as light sources in long-distance and large-scale sensing systems. Section 3 and Section 4 provide recommendations on the system performance of RFLs based on Rayleigh scattering and grating feedback as sensing elements, respectively. Finally, the research progress in recent years is summarized, and the future development directions in recent years are explored.

## 2. RFL as a Light Source for the Sensing System

The feedback of RFL based on SMF only relies on the inherent Rayleigh scattering in the fiber; additionally, the Rayleigh scattering along the cavity is equivalent to multiple or even countless random weak mirrors, averaging the frequency noise. Therefore, RFL exhibits astonishing low-frequency noise characteristics [51,52,53,54], which has an absolute advantage in high-performance fiber optic sensing systems. The performance of the laser light source is crucial for fiber optic sensing systems; for example, the output stability, linewidth, and noise performance play a decisive role in the detection distance, signal-to-noise ratio, resolution, and accuracy of fiber optic sensing systems. Furthermore, in systems without any added point reflectors, the generated random laser spectrum, power, and other stabilities are good and are not sensitive to external temperature changes [55]. The excellent thermal stability prevents the introduction of noise environment interference, which is conducive to improving the measurement accuracy and signal-to-noise ratio performance of the sensing system; this shows great advantages in ultra-long-distance sensing. Similarly, various point reflectors can be added to the RFL system to achieve sensing of different parameters (temperature, strain, ultrasonic sensing, etc.) by changing the information of the external environment, such as fiber Bragg grating (FBG), Fabry–Perot cavity [56], Sagnac ring mirror [57], artificially controlled backscattering fiber reflectors [58], and a resonator composed of two couplers [59]. More importantly, point reflectors can dissociate the demodulator for a long distance (over a hundred kilometers) and are easy to combine with various multiplexing techniques; this shows potential for constructing large-scale and long-distance sensing networks.

### 2.1. High-Resolution Sensing System

**Figure 2:**
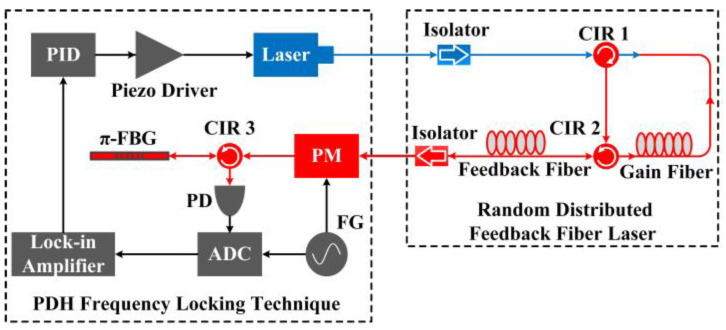
Experimental setup of RFL. Reprinted/adapted with permission from Ref. [61]. Copyright 2018, copyright Optics Letters.

**Figure 3:**
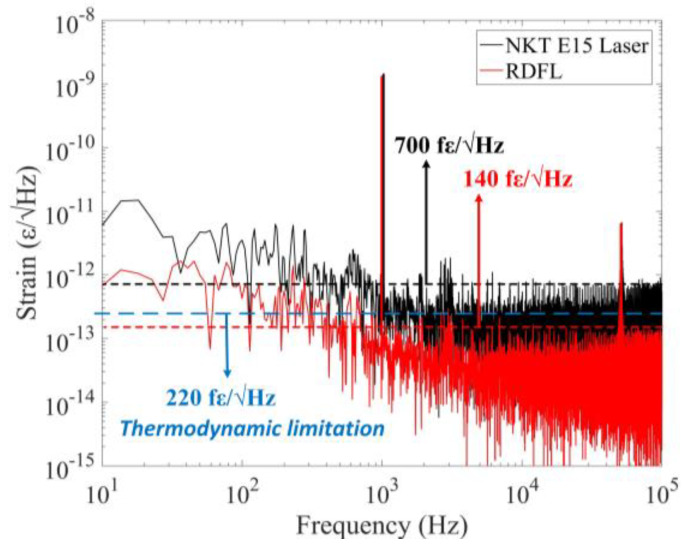
Strain power spectral density of two lasers. Reprinted/adapted with permission from Ref. [61]. Copyright 2018, copyright Optics Letters.

Due to the unique structure of RFL, an important advantage in the sensing field is its narrow linewidth and low noise, and by combining high-precision demodulation technology, high-resolution signal detection can be achieved and has been effectively confirmed in previous research. The Public University of Navarra [60] combined FBG and phase-shifted fiber Bragg grating (π-FBG) as sensing elements, achieving a high-resolution sensing system with a temperature resolution of less than 0.01 °C and a strain resolution of less than 0.2 με. The Semiconductor Research Institute of University of the Chinese Academy of Sciences [61] separated the feedback fiber and the gain fiber, and introduced the π-FBG as the strain sensing element. Combined with the frequency-shifted Pound–Drever–Hall technology, which tracked the resonant frequency change of the π-FBG, the frequency noise of the laser at 1 kHz decreased from 100 Hz/Hz^1/2^ to 20 Hz/Hz^1/2^ and achieved 140 fε/Hz^1/2^ with ultrahigh dynamic strain resolution, as shown in Figure 2 and Figure 3. This value was very close to the theoretical value of 130 fε/Hz^1/2^, which fully demonstrated the advantages of random fiber lasers in the field of high-resolution dynamic sensing.

### 2.2. Long-Distance Sensing System

**Figure 4:**
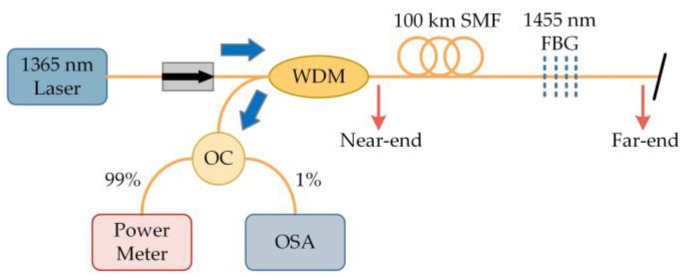
Experimental setup of the RFL with open cavity.

**Figure 5:**
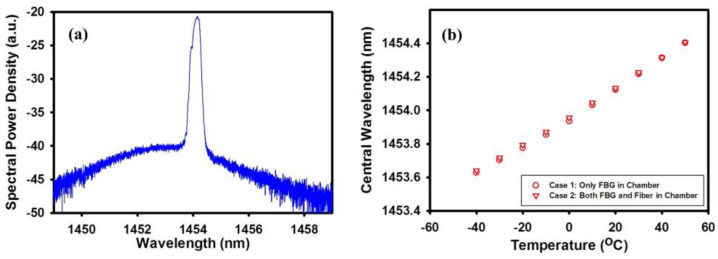
(**a**) Output spectrum in room temperature; (**b**) temperature response of the lasing. Reprinted/adapted with permission from Ref. [55]. Copyright 2012, copyright Optics Express.

**Figure 6:**
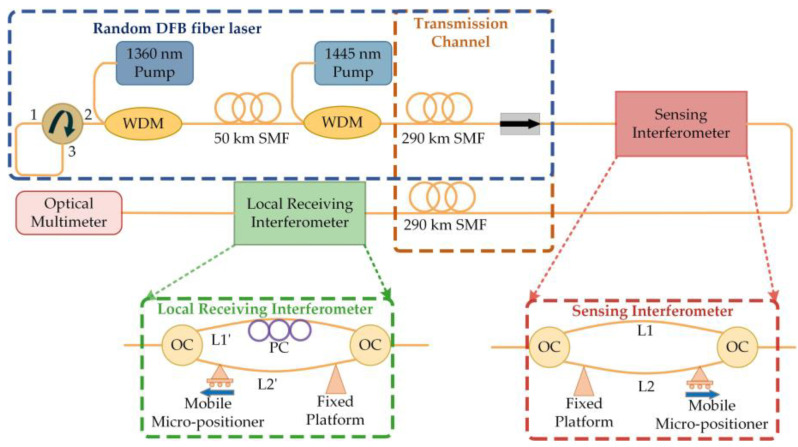
Experimental setup of the RFL with a Mach–Zehnder sensor.

**Figure 7:**
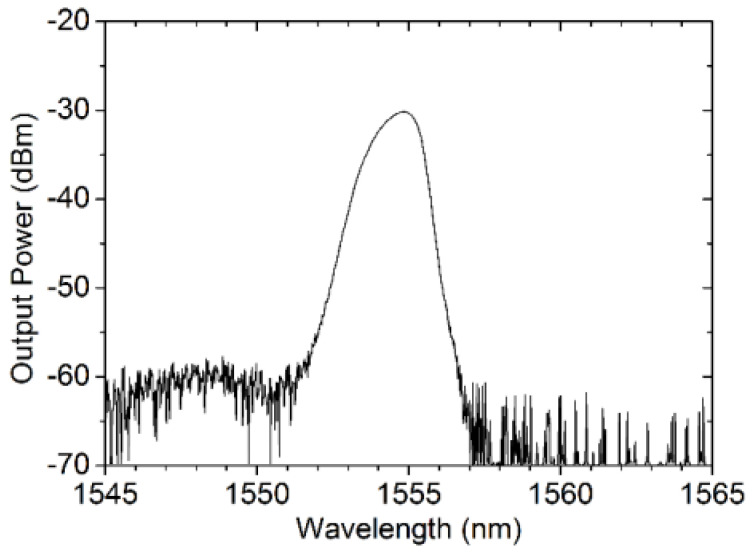
Optical spectrum of RFL measured after 290 km. Reprinted/adapted with permission from Ref. [64]. Copyright 2018, copyright Optics Express.

**Figure 8:**
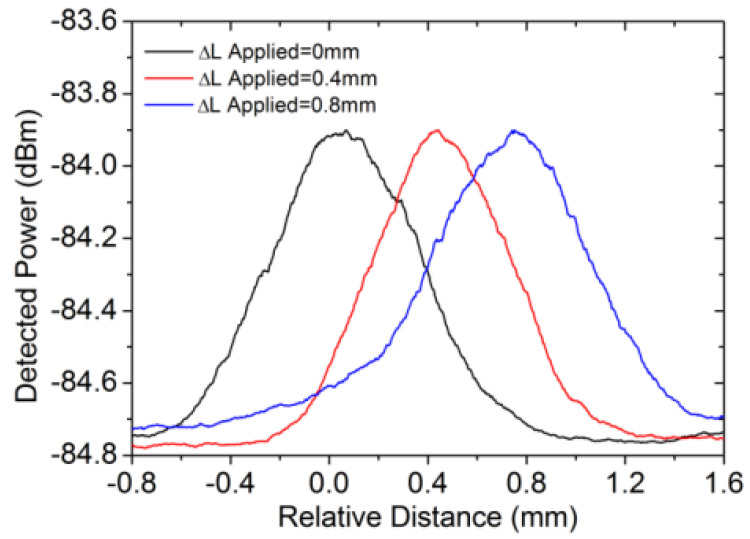
Experimental traces detected of the ocal receiving interferometer. Reprinted/adapted with permission from Ref. [64]. Copyright 2018, copyright Optics Express.

In recent years, the characteristics of fiber optic sensing systems that can achieve ultra-long-distance and ultra-large-range monitoring without a power supply to sensors have attracted much interest. Unfortunately, signal transmission with fiber optic transmission is accompanied by loss. Although low-loss fibers are generally selected as transmission channels, when the distance increases to hundreds of kilometers, enormous losses will also accumulate, and the SNR of the signal will also be greatly reduced. To achieve accurate and long-distance sensing, optical amplifiers have become essential devices, which greatly increases the cost and complexity of the system. New concepts using RFL can potentially be used to solve this problem. The stable and balanced distributed amplification and long-cavity characteristics of RFLs are particularly suitable for ultra-long-distance applications, and since amplifiers are not needed, signal transmission of hundreds of kilometers can be achieved, with the potential for long-distance relay-free transmission. For example, the University of Electronic Science and Technology of China [55,62] used a 100 km SMF as the feedback and gain amplification medium, and the pump was launched into the fiber and the point reflector FBG at the end through a wavelength division multiplexer, as shown in Figure 4. Due to the mode selection effect of FBG, RFL exhibited stable single-wavelength output, as shown in Figure 5a. By placing FBG, FBG, and 100 km fiber into the temperature control chamber, respectively, the measurement results matched well, as shown in Figure 5b, which indicated that the distributed feedback fiber was temperature insensitive, and only FBG was temperature sensitive. The good thermal stability of RFL effectively avoided external environmental interference and achieved long-distance sensing with an SNR of >20 dB and a transmission distance of >100 km, indicating the superiority of RFL in long-distance sensing systems. Furthermore, by utilizing ultra-low-loss fiber (ULLF, G.654.E, Yangtze Optical Fiber and Cable Joint Stock Limited Company, Wuhan, China) with Rayleigh backscattering coefficient and Raman gain coefficient lower than SMF (G.652.D) [63], the ULLF had an attenuation coefficient of 0.16 dB/km at 1550 nm, while SMF had an attenuation coefficient of 0.175 dB/km. The Rayleigh backscattering coefficient and Raman gain coefficient were 1 dB and 1.7 dB lower than SMF, respectively. The intracavity laser could be moved further away, breaking through the limitation of RFL transmission length and extending the length to 200 km. The Public University of Navarr [64] extended the RFL to low-coherence fiber optic sensing systems, as shown in Figure 6; the distributed feedback and amplification were provided by SMFs of 50 km + 290 km. At the end of the cavity, in front of the sensing interferometer, an isolator was placed to prevent any reflection. The transmission channel was composed of two identical 290 km SMFs, and the fiber optic low-coherence interferometry was composed of two Mach–Zehnder interferometers. By moving the mobile micropositioner, displacement was applied to one arm of each of the two interferometers (L2 and L2′). The detected interference signal depended on the polarization of the two signals. Therefore, a polarization controller (PC) was connected to the other arm (L1′) of the local receiving interferometer to maximize the visibility of the interference fringes. After passing through 290 km fiber, the spectrum of the system, shown in Figure 7, reached a peak power of −30.18 dBm. Figure 8 shows the signal detected after applying displacement, and the displacement of the center fringe position was clearly discernible, which indicated that the changes of the sensing interferometer could be accurately obtained. There was no crosstalk in the system, which further demonstrated the ability of RFL for remote sensor monitoring.

### 2.3. Large-Scale Sensing System

**Figure 9:**
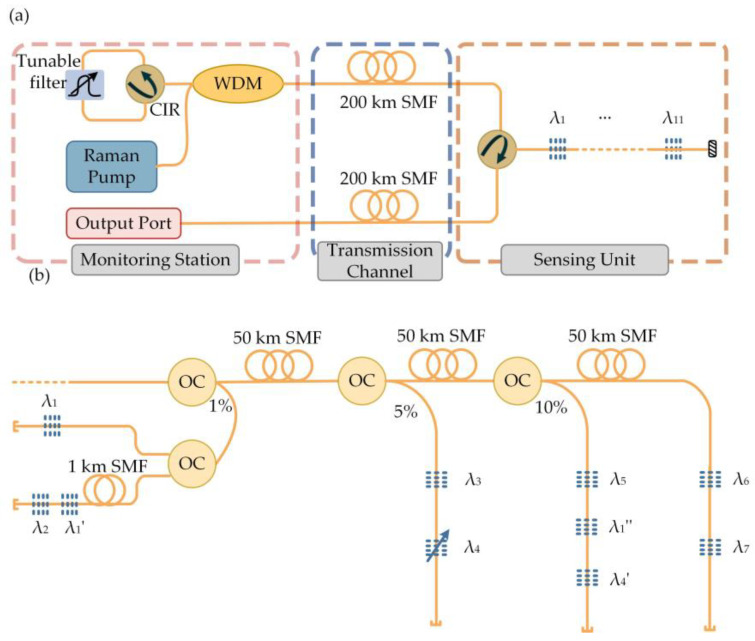
(**a**) Experimental setup of the RFL; (**b**) experimental setup of the sensor network.

A traditional remote sensing network uses a broadband light source; when the system distance is greater than 25 km [65], noise introduced by Rayleigh scattering and loss along the fiber are inevitable, and the signal detectability deteriorates. RFL makes use of Rayleigh scattering in the fiber; thus, the transmission distance is no longer limited. Moreover, RFLs have previously been shown to achieve sensing for hundreds of kilometers without the need for amplification. The most important aspect is that the structure of the RFL combined with point reflectors is highly convenient for building a transmission network. Through technical means, such as wavelength division multiplexing (WDM), time division multiplexing (TDM), and space division multiplexing (SDM), a sensing network is constructed in a system of hundreds of kilometers, greatly reducing the cost of the system. The Public University of Navarra [66] reused 11 FBGs for sensing monitoring in a 200 km RFL system using WDM technology, as shown in Figure 9a. The first reflector of RFL was composed of a circulator and a tunable filter, the second was based on backward Rayleigh scattering of SMF, and 11 FBGs were connected to the transmission channel through a circulator to ensure unidirectional laser operation. It could be used to monitor FBG beyond 200 km by adjusting the wavelength of the tunable filter. Similar results were achieved using TDM and WDM techniques in a 200 km system [67], achieving a sensing network of 10 FBGs, as shown in Figure 9b. Different types of gain fibers or wavelength tunable pumps can be used to control the gain curve, achieving wider spectra and more FBGs multiplexing [68]. RFL combined with FBG has enormous development potential in achieving ultra-long-distance sensing systems. In the future, it can be combined with other point reflectors or multiplexing methods to reduce system costs while further improving system performance and expanding the sensing network.

Due to its good stability and low noise characteristics, the RFL is a suitable light source for high-resolution and long-distance sensing systems, achieving high-performance sensing. In long-distance sensing networks, hundreds of kilometers of transmission distance can be achieved without optical amplifiers, the SNR is high, and the signal quality is good. By combining multiple point reflectors to construct a remote sensing network, the cost and complexity of the system are greatly reduced, causing it to be an ideal long-distance fiber optic sensing system. It is anticipated that a longer-distance sensing network based on random fiber lasers will be attained soon.

## 3. Rayleigh Scattering-Based RFL as a Sensitive Element

A point-type sensor was described in Section 2; in a point-type sensor, one or more sensors are placed at the end of the RFL. In this section, the feedback fiber is directly used as the sensing fiber, which is equivalent to distributing countless sensors on a single fiber, achieving high spatial resolution measurement. This is of great significance for areas such as perimeter security, oil and gas pipelines, and bridge tunnel monitoring. In general, the distributed fiber optic sensing measurement distance needs to be long, with a high spatial resolution and high SNR, to achieve high accuracy and real-time measurement. In practical engineering applications, various limiting factors are present. For example, in Brillouin optical time-domain analysis (BOTDA), the SNR is reduced due to the fiber loss involved in the transmission process, and the detection distance and measurement accuracy are mutually constrained. Achieving long-distance, high spatial resolution, and accuracy is a major challenge faced by BOTDA [69]. The unique properties of gain flatness, good stability, and low noise of RFL are expected to overcome this challenge. This section focuses on the research progress of RFLs in the field of distributed sensing.

**Figure 10:**
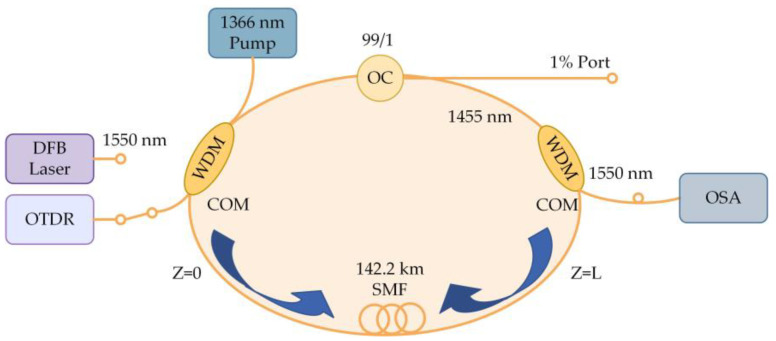
Experimental setup of a ring cavity RFL.

**Figure 11:**
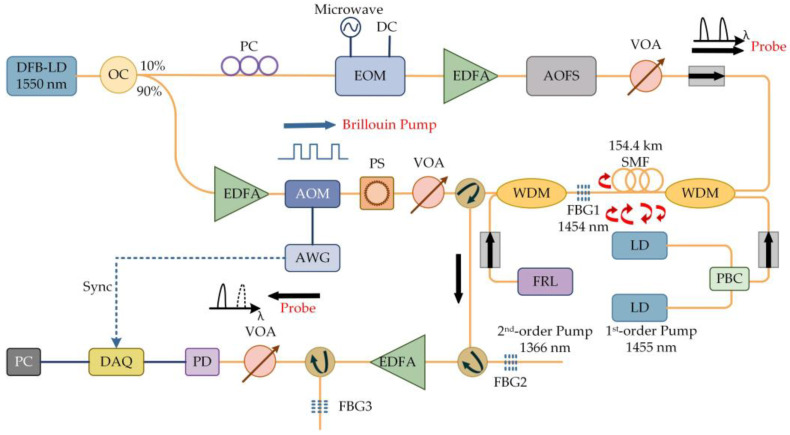
Experimental setup for long-distance BOTDA pumped by combining a random laser and first-order low-noise LD.

**Figure 12:**
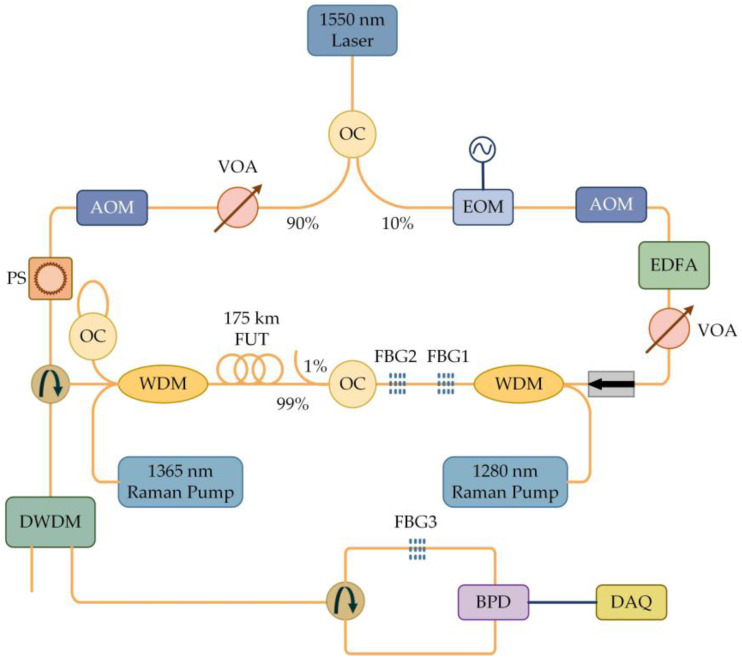
Experimental setup for second-order and third-order hybrid amplification of RFL.

The first experiment by the University of Electronic Science and Technology of China demonstrated the application prospect of the RFL in the field of distributed sensing [70]. The RFL’s distributed Raman amplification and optical pulse coding technology were combined, and the experimental setup is shown in Figure 10. The light emitted by a 1550 nm laser was injected into SMF through a WDM, and the output spectrum was monitored using an optical spectrum analyzer (OSA). This system had a sensing distance of over 142.2 km, a spatial resolution of 5 m, a temperature accuracy of ±1 °C, and the sensing distance record of the no-relay BOTDA at this time was attained at 93 km [71]; this successfully achieved an improvement in the BOTDA sensing distance. However, the low Rayleigh scattering coefficient of RFL led to a relatively high lasing threshold, which could easily introduce nonlinear effects and cause spectral broadening and was very detrimental to further improving the performance of the system. To increase the Rayleigh backscatter intensity and reduce the threshold, the refractive index of the fiber core can be changed by adjusting the concentration of germanium doping [72] or using high nonlinear optical fibers [73]. Furthermore, the low noise characteristics of RFL also facilitated a new direction to overcome this difficulty. By combining a low-noise laser-diode (LD) pump and RFL second-order pump [74], a hybrid pump structure was formed, which greatly aided in the improvement of the pump efficiency and the reduction in the power and nonlinear effects, as shown in Figure 11. The light emitted by the LD was divided into two parts through a coupler, and 10% of the light was generated into two side-bands through an electro-optic modulator (EOM). It was amplified by an erbium-doped fiber amplifier (EDFA) and entered an acoustic-optic frequency shifter (AOFS). After passing through a variable optical attenuator (VOA), optical isolator, WDM, and FBG, it reached the sensing fiber. The additional 90% of the light was amplified by EDFA as a Brillouin pump. The coding was achieved by an acousto-optic modulator (AOM) driven by an arbitrary waveform generator (AWG). By using a polarization scrambler (PS) to reduce polarization-related gain fluctuations, the Brillouin pump entered the sensing fiber through the VOA, circulator, and WDM. The receiver was composed of an EDFA, two circulators, two FBGs, a photodetector (PD), and a data acquisition card (DAQ). The sensing distance was further increased to 154.4 km without sacrificing spatial resolution and temperature measurement accuracy, which again set a new record for BOTDA. Similarly, the sensing distance was further improved (175 km) by using a mixed amplification method of second order and third order [75], and the experimental setup is shown in Figure 12. The light emitted by the laser was divided into 90% pulse light and 10% probe light by the coupler. An EOM was used to modulate the continuous light into a double-sided band probe light to reduce the influence of dual pumping on relative intensity noise transfer. A 175 km SMF was used for distributed amplification and feedback; at the time, this was the longest no-relay sensing distance for BOTDA internationally.

**Figure 13:**
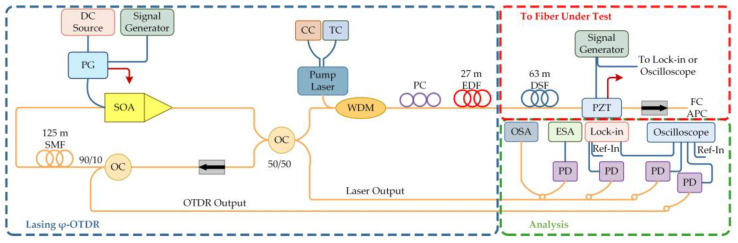
Experimental setup of the φ-OTDR.

The advantages of low noise and gain equalization of the RFL for improving the performance of the phase-sensitive optical time-domain reflectometer mode (φ-OTDR) are also of great help. In traditional φ-OTDR systems, with the extension of sensing distance, the signal strength exponentially decreases, causing extreme difficulty in long-distance signal detection. The Pontifical Catholic University of Rio de Janeiro [76] used Rayleigh scattering of dispersion shifted fiber (DSF) to provide random distribution feedback, and the backscattered light was synchronously amplified in the laser cavity, as shown in Figure 13. The pump was regulated by a current controller (CC) and a temperature controller (TC), and an erbium-doped fiber (EDF) was inserted into the fiber loop to provide bidirectional optical amplification. By driving a semiconductor optical amplifier (SOA) through a pulse generator (PG), laser pulses were obtained from SOA and the Rayleigh backscattered light returned by the DSF. A piezoelectric transducer (PZT) was used to realize vibration sensing measurement. The spatial resolution was 1.37 m, the measurement distance was 63 m, and the SNR was 23 dB. It could measure vibrational signals along the fiber optic cable and was used for perimeter security, seismic wave detection, and oil pipeline safety detection, which had important economic and social significance.

At present, the RS-RFL has achieved good results in improving the performance of distributed sensing systems; for example, the sensing distance and the spatial resolution have broken records multiple times, fully demonstrating the application prospects of RFL in the field of distributed sensing, which is of great help for the safety monitoring of important national facilities, such as bridges and tunnels. In future research, efforts can be performed to address the temperature and strain cross-sensitivity issues of BOTDA systems and RFL for other distributed sensing technologies to further expand the RFL application fields.

## 4. Grating Feedback-Based RFL as a Sensitive Element

The difference between grating feedback-type random fiber lasers and Rayleigh scattering-type RFLs lies in the different media used to provide feedback. This type of RFL mainly involves engraving randomly distributed weak fiber grating arrays (RFGA) on SMFs or active fibers, providing feedback through photon localization effects. Scattered light returns to the origin after multiple scattering to form a closed loop [77,78,79], and the laser exhibits high directionality and sharp peak related to the high Q value of the resonant cavity. Compared to RS-RFL, RFGA significantly enhances the feedback strength, resulting in a significant reduction in fiber length and threshold. Moreover, the multiple gratings in RFGA are equivalent to Fabry–Perot cavities with different cavity lengths, and the filtering effect helps to suppress mode competition. Therefore, the laser spectral line width is further narrowed and the frequency noise and relative intensity noise are also reduced [80], which creates advantages for high-precision fiber optic sensing. However, because there may be more than one grating pair that meets the resonance condition among numerous weak gratings, the output stability of the laser is affected. Generally, a matched filter can be added to the system for frequency stabilization while further reducing the line width and noise, which is extremely helpful for high-precision fiber optic sensing. This section mainly reviews the research progress of this type of RFL in the sensing field in recent years from two aspects: RFGA and narrowband filters as sensitive components.

### 4.1. Random Weak Grating Array as a Sensitive Element

Since RFGA is generally composed of many randomly spaced weak gratings or numerous randomly spaced scattering enhancement points, the reflection spectrum is wide, and multiple refractive index modulation points are equivalent to randomly spaced Fabry–Perot cavities, resulting in multiple random modes. At the same time, the refractive index changes and dispersion effects vary at different positions, resulting in differences in temperature and strain characteristics, creating favorable conditions for multiparameter sensing. The combination of the characteristics of lower noise and narrower linewidth in this type of RFL also has broad prospects for improving the resolution and accuracy of sensing systems and has attracted the attention of many scholars in the field of fiber optic sensing. This section focuses on the research progress of the RFLs in the field of point sensing.

**Figure 14:**
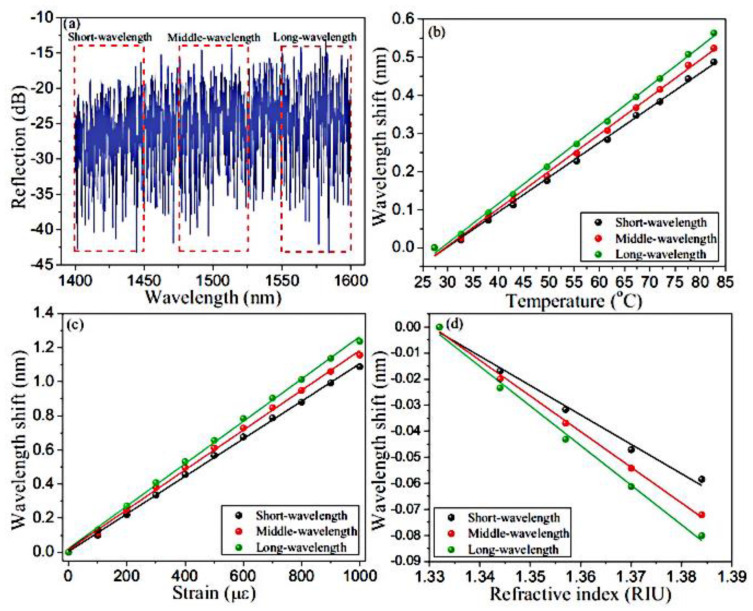
(**a**) Spectra of the selected 3 positions; test results of (**b**) temperature, (**c**) strain, and (**d**) refractive index. Reprinted/adapted with permission from Ref. [82]. Copyright 2015, copyright Optics Letters.

**Figure 15:**
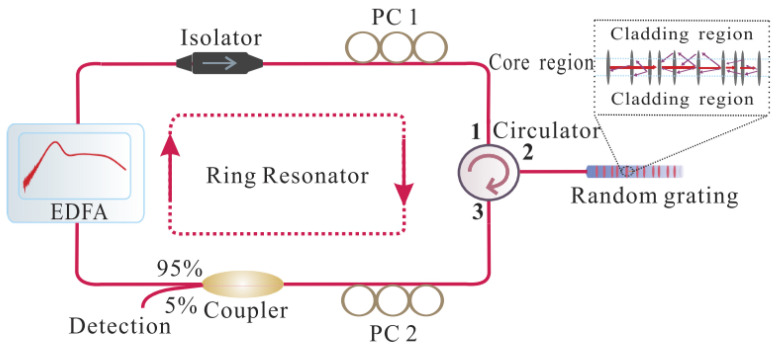
Experimental setup of RFL based on EDFA. Reprinted/adapted with permission from Ref. [83]. Copyright 2016, copyright AIP Advances.

**Figure 16:**
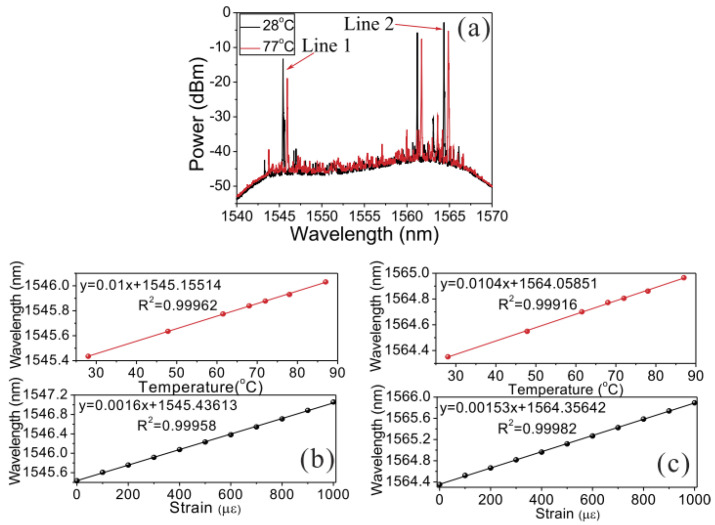
(**a**) Output spectrum at different temperatures; (**b**) temperature and strain measurements of Line 1; (**c**) temperature and strain measurements of Line 2. Reprinted/adapted with permission from Ref. [83]. Copyright 2016, copyright AIP Advances.

RFLs based on RFGA have attracted scientific attention worldwide for their unique advantages. By designing different RFGAs, such as RFGAs with randomly varying refractive indices, the needs of multiparameter measurement can be met. The measurement of temperature, strain, and refractive index has been previously studied. With an etched-core FBG [81], simultaneous measurement is achieved by utilizing the difference in the wavelength shift of excited higher-order modes under different parameters. However, precise control of the etching time and speed is needed, the preparation of the sensor is cumbersome, and the mechanical strength of the sensor is reduced after etching. RFGA does not require special processing and can achieve simultaneous measurement of three parameters by monitoring wavelength shifts in different wavelength regions, with simple operation and high physical strength. The University of Ottawa prepared 50,000 refractive index modulation points on a 10 cm standard single-mode fiber using a femtosecond (fs) laser. The cross-correlation wavelength drift at three positions was monitored by an OSA [82], achieving recognition of temperature, strain, and refractive index, with sensitivities of 10.32 pm/°C, 1.24 pm/με, and −1520.6 nm/RIU, as shown in Figure 14. Furthermore, RFGA was used as a feedback component and combined with an erbium-doped fiber amplifier to provide gain [83], and the experimental setup is shown in Figure 15. The output multiple laser lines had an SNR of up to 40 dB. By utilizing laser lines at different positions to respond differently to the environment, simultaneous measurement of temperature and strain was also achieved, as shown in Figure 16. Among them, the temperature sensitivities of Line 1 and Line 2 were 10 pm/°C and 10.4 pm/°C, and the strains were 1.6 pm/με and 1.53 pm/με, respectively; these have broad development prospects in the fields of biomedicine and environmental sensing.

**Figure 17:**
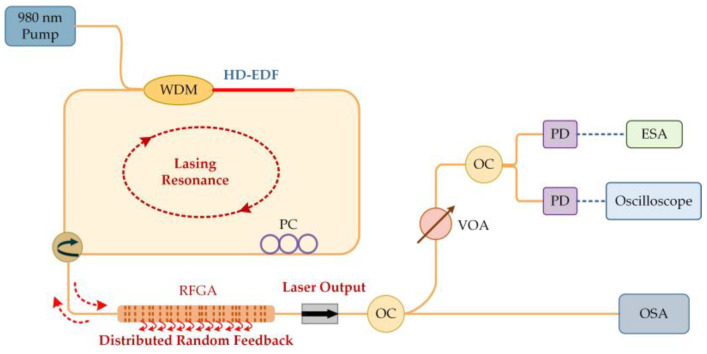
Experimental setup of RFL based on random fiber gratings.

**Figure 18:**
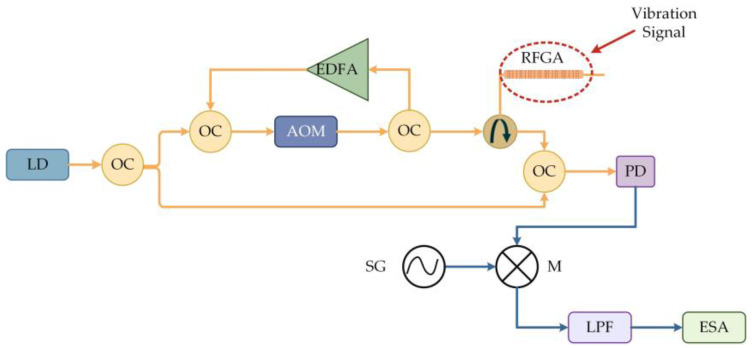
Self-heterodyne detection system based on random gratings.

The low noise and narrow linewidth characteristics of RFL are expected to achieve high-resolution ultrasound measurement; this achievement would be of great significance for the fields of structural health detection and geophysical exploration where the waiting signal is extremely weak. An evident phenomenon of generating ultrasounds is acoustic emission, where energy is released in the form of elastic stress waves, containing a large amount of information related to damage. The frequency of elastic waves is generally in the range of several kHz to several MHz, and the response amplitude is in the hundreds of nε and sub-pε; thus, the sensor needs to have a wide detection range and high sensitivity and resolution. Traditional FBG has a wide reflection bandwidth, and its sensitivity is limited by the broadband light source [84], causing difficulty to improve the resolution. The University of Ottawa used fs laser-engraved RFGA as an ultrasound-sensitive probe [85] and utilized the wide reflection spectrum of RFGA. The laser ultrasound sensor had a linear response to ultrasound over a wide frequency range, without the need for strict setting of operating points. Compared with traditional piezoelectric transducer sensors, the response light SNR was 20 dB higher, and the sensitivity was four times higher in the broadband ultrasound range of 20 kHz~0.8 MHz. After optimizing the production process of the RFGA, the scattering intensity of the RFGA increased by 13.5 dB. Combined with high-gain erbium-doped fiber, the frequency band was extended to 8 MHz, and the SNR was still greater than 43 dB at 5.1 MHz. The experimental setup is shown in Figure 17 [86]. RFGA was introduced into the loop by a circulator. Similarly, RFGA-based ultrasonic testing had a high resolution, which was calibrated by combining self-heterodyne acousto-optic frequency comb interrogation technology and a heterodyne interferometer [87], as shown in Figure 18. An AOM generated an acousto-optic comb, which was amplified by an EDFA, and the comb was injected into RFGA through a circulator. The backward reflection of RFGA was mixed with part of the LD light. Finally, a PD was used to detect the output light; this achieved 114 pε signal detection, which was of great significance for structural health monitoring, crack detection, and nondestructive evaluation of nε magnitude. However, a certain gap from the sub-pε magnitude was still present.

RFL based on RFGA has been widely studied in temperature, strain, and ultrasonic detection. By utilizing different grating sensitivities at different positions, multiparameter sensing can be achieved. Compared to traditional measurement systems that combine different gratings [88,89,90], the sensor-based RFGA reduces complexity and cost and improves mechanical strength. The strong random scattering characteristic of RFGA is also able to solve the problem of traditional FBG being limited by broadband light source, which causes difficulty to achieve high-resolution monitoring. The wide spectral characteristics also make the sensor effective within this wavelength range, and the sensor is suitable for harsh environments with extreme temperatures and strain. Moreover, the advantages of narrow line width and low noise are also beneficial for improving sensitivity and resolution, which has great guiding significance for ultrasonic detection.

### 4.2. Narrowband Filter in RFL as Sensitive Element

The RFL based on RFGA has poor output stability due to the large number of grating pairs that meet resonance conditions. To improve the stability of the RFL, a narrowband filter can be added to the system for frequency stabilization. Due to the filtering effect, the stability of RFL is greatly improved, and the line width and noise are further reduced. This has great advantages in improving the resolution of fiber optic sensing systems, especially strain sensing. This section primarily introduces the progress of using narrowband filters as sensitive elements.

**Figure 19:**
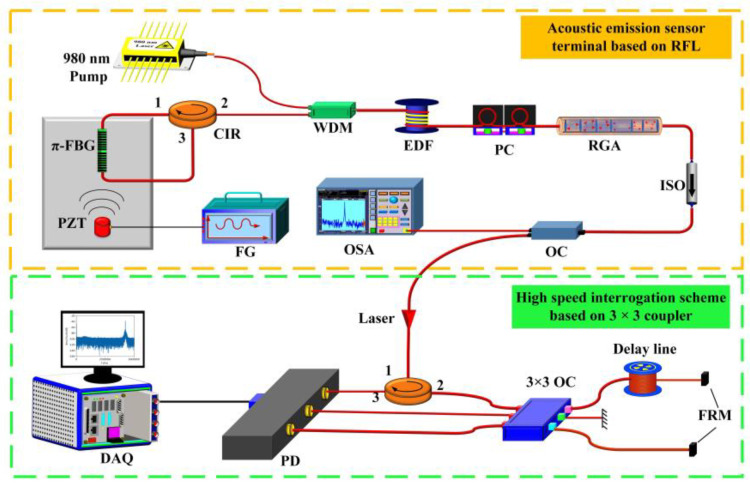
Experimental setup of RFL for acoustic emission detection. Reprinted/adapted with permission from Ref. [92]. Copyright 2020, copyright Optics Express.

**Figure 20:**
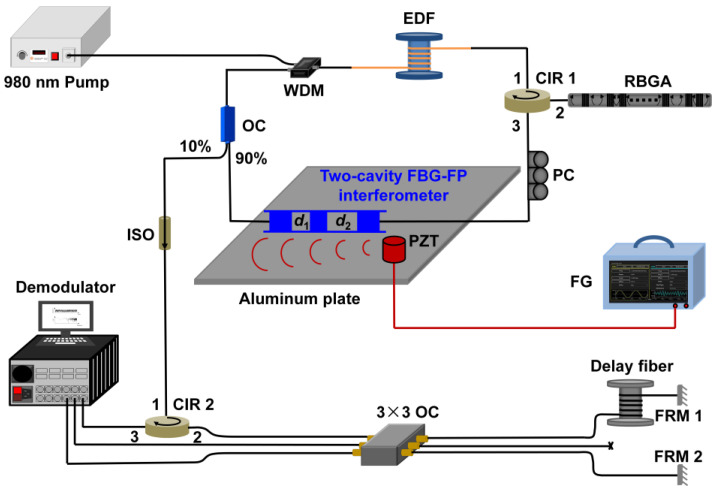
Experimental setup for high dynamic strain resolution detection. Reprinted/adapted with permission from Ref. [93]. Copyright 2022, copyright IEEE Photonics Technology Letters.

**Figure 21:**
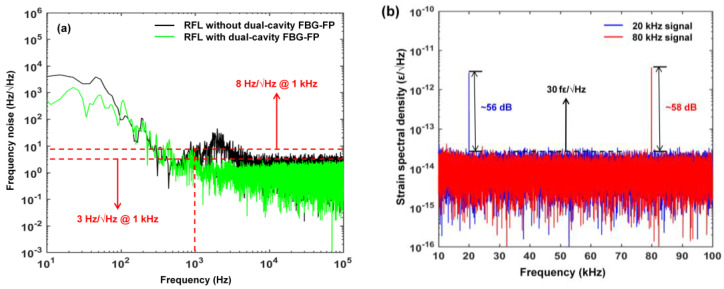
(**a**) Frequency noise measurement of the proposed RFL with or without the dual-cavity FBG-FP; Reprinted/adapted with permission from Ref. [94]. Copyright 2022, copyright Journal of Lightwave Technology;(**b**) power spectral density of the measured dynamic strain signal. Reprinted/adapted with permission from Ref. [93]. Copyright 2022, copyright IEEE Photonics Technology Letters.

In Section 4.1, we mentioned using RFGA as an ultrasound-sensitive probe, and although good results have been achieved with improved dynamic resolution on the order of hundreds of pε, they are still unsatisfactory for a number of applications where sub-pε signal monitoring is needed. After adding a narrowband filter to the system, the line width and frequency noise can be greatly reduced, and a higher-resolution acoustic detection is expected [91]. The Institute of Semiconductors of the Chinese Academy of Sciences introduced a π-FBG as a frequency selection element in the RFL system. The experimental setup is shown in Figure 19. A 980 nm pump was injected into the EDF through WDM. The 30 equivalent weakly reflective FBGs prepared by ultraviolet exposure technology provided feedback. The PC was used to adjust the polarization state of the laser, and the output spectrum was monitored in real time through OSA. The π-FBG was placed on an aluminum plate as an acoustic emission probe, and 3 × 3 coupler interrogation technology was used for demodulation. The 3 dB bandwidth of π-FBG was GHz, the filtered RFL linewidth was approximately 548 Hz, and the frequency noise was 10 Hz/Hz^1/2^ @ 1 kHz. When the π-FBG was used as an acoustic emission detection element [92], the resolution reached 280 fε/Hz^1/2^ @ 1 kHz, which could already meet most high-precision measurement needs. Similar results were obtained by replacing the π-FBG with a dual-cavity fiber grating Fabry–Perot interferometer [93,94,95], as shown in Figure 20. The Fabry–Perot cavity interferometer suppressed multipeak laser modes and frequency jitter, reducing frequency noise to 3 Hz/Hz^1/2^, as shown in Figure 21a. The dynamic strain resolution increased to 30 fε/Hz^1/2^, which was close to the theoretical value of 19.8 fε/Hz^1/2^. The response of the RFL sensor to dynamic strain signals is shown in Figure 21b, and the strain signal was effectively restored. The introduction of a narrow bandwidth filter in the system helped to achieve a narrow linewidth and low-noise RFL and improved the dynamic strain resolution to fε; this result indicated that the RFL based on RFGA had significant advantages in high-resolution dynamic strain monitoring systems.

**Figure 22:**
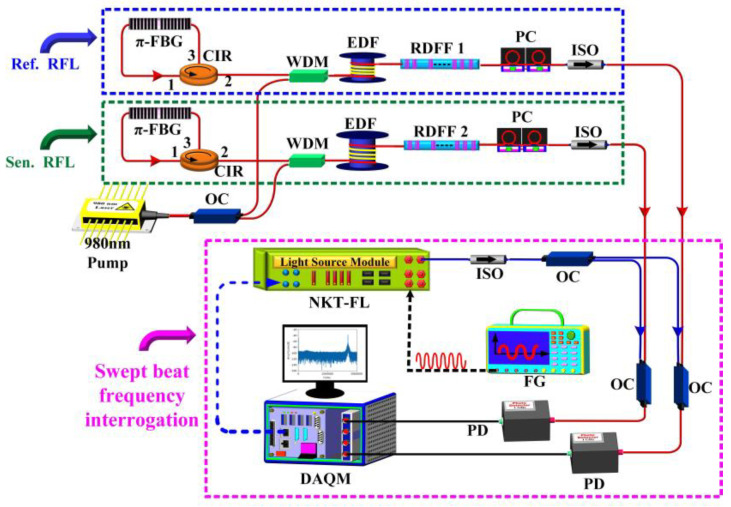
Demodulation structure diagram of a static strain sensor based on RFL. Reprinted/adapted with permission from Ref. [96]. Copyright 2019, copyright IEEE Photonics Technology Letters.

The RFL also provides a new approach for high-resolution static strain sensing. Unlike dynamic strain sensing, static strain sensing is often affected by external environmental disturbances and low-frequency noise, generally on the order of nε. By introducing π-FBG into RFL for filtering and using an identical reference RFL to compensate for the frequency drift of the scanning laser caused by temperature (the experimental setup is shown in Figure 22), the demodulation was realized through sweep-beat frequency technology. A static strain resolution of 196 pε was achieved [96], which was of great value for applications in the fields of oil and gas exploration and crustal deformation.

At present, π-FBG and Fabry–Perot cavities are mainly used for mode regulation, and a dynamic strain resolution of fε magnitude is obtained. Compared with the pε magnitude of traditional fiber optic laser sensors [97], the measurement level demonstrates a qualitative leap, which is of crucial significance for fields such as geophysical exploration and structural health monitoring. However, research has mostly focused on dynamic strain sensing, while research on static strain is relatively limited. This is mainly due to the low noise of the RFL in the high-frequency range, which is easily affected by external environmental disturbances in the low-frequency range and results in high noise and limited application.

## 5. Summary and Outlook

In the past decade, researchers have proposed various RFL structures, achieving narrow linewidth and low-noise laser output. RFL has been extended to practical applications, especially in the field of fiber optic sensing. RFL is used as a light source or sensing element to apply temperature, strain, or acoustic emission signals, achieving long-distance, high SNR, and high-resolution sensing; the RFL can be applied for structural health detection, perimeter security, and bridge tunnel monitoring and provides high-performance light sources or sensors, and these applications promote RFL development and enrich the research content of RFL.

Although RFLs have achieved valuable results in the field of fiber sensing, many avenues of research are available to explore. For example, the cross-sensitivity between temperature and strain in a system based on the combination of RFL and BOTDA needs to be addressed. For high-precision static strain sensing systems, low-frequency RFL noise needs to be suppressed. The development of RFL in the sensing field also needs further exploration, such as using RFGA to achieve multiparameter sensing at the mm-level resolution within a range of tens of meters, for use in pipeline leakage detection and microseismic monitoring fields. Combined with its advantage of low noise, RFLs have been used in hydrophones to improve the minimum detectable level or expand the detection range; additionally, RFLs promote the development of underwater target location, identification and tracking, marine oil and gas resource exploration, submarine earthquake monitoring, and other fields. Developing flexible RFLs for structural attitude monitoring of aerospace vehicles, robot shape perception, and trajectory tracking of minimally invasive surgical catheters and probes can provide flexible sensors with high sensitivity and spatial resolution.

## Data Availability

No data are associated with this research work.
